# Genetically predicted serum Pimelylcarnitine mediates the association between CD39^+^ secreting Treg cells and intervertebral disc degeneration

**DOI:** 10.1097/MD.0000000000048540

**Published:** 2026-05-15

**Authors:** Na Li, Cimin Shen, Zhenv Huang, Zhuoyi Shao, Jietao Wu, Danyun Hua, Lu Wang

**Affiliations:** a Department of Acupuncture and Moxibustion, Fenghua Hospital of Traditional Chinese Medicine, Ningbo, Zhejiang, China.

**Keywords:** CD39^+^, intervertebral disc degeneration, Mendelian randomization, pimelylcarnitine, regulatory, T-lymphocytes

## Abstract

Investigating the causal relationship between CD39^+^ secreting Treg cells and intervertebral disc degeneration (IVDD) and identifying and quantifying the role of pimelylcarnitine (C7-DC) as a potential mediator. Using summary-level data from a genome-wide association study, a two-sample Mendelian randomization (MR) analysis of genetically predicted CD39^+^ secreting Treg (N = 3045) and IVDD (41,669 cases, 294,770 controls) was performed. Furthermore, two-step MR was used to quantitate the proportion of the effect of C7-DC protein-mediated RA on IVDD. MR analysis identified higher genetically predicted CD39^+^ secreting Treg on immune cells (primary MR analysis odds ratio (OR) 1.02/SD increase, 95% confidence interval [CI]: 1.01–1.03) increased risk of IVDD. There was no strong evidence that genetically predicted IVDD had an effect on CD39^+^ secreting Treg (OR: 0.58, 95% CI: 0.23–1.49). The proportion of genetically predicted IVDD mediated by C7-DC was 9.1%. In conclusion, the present study identifies a causal relationship between CD39^+^ secreting Tregs in immune cells and IVDD. A small proportion of the effect is mediated by C7-DC, whereas the majority of the mechanism underlying the effect of CD39^+^ secreting Tregs on IVDD remains unclear. These findings may provide novel therapeutic targets and offer valuable insights for further investigations into the underlying mechanisms.

## 1. Introduction

Intervertebral disc degeneration (IVDD) is a prevalent and complex degenerative disorder characterized by progressive deterioration in the structure and function of the intervertebral disc, which ultimately leads to chronic low back pain, motor dysfunction, and a marked decline in patients’ quality of life.^[1]^ With the accelerating global population aging, the prevalence of IVDD continues to rise, making it a major public health issue responsible for disability and substantial consumption of medical resources.^[2]^ Current therapeutic approaches mainly focus on symptomatic relief, including physical therapy, pharmacological intervention, and surgical treatment. Nevertheless, these strategies often fail to effectively halt the pathological progression, and are limited by unstable efficacy, long recovery periods, and potential complications.^[3]^ Therefore, in-depth exploration of the pathogenesis of IVDD, particularly its molecular immunological basis, is of great significance for the development of targeted preventive and therapeutic strategies.

In recent years, the role of the immune system in the pathogenesis of IVDD has attracted increasing attention. Abnormal activation of host immune cells and imbalance of inflammatory mediators are considered key drivers of IVDD.^[4,5]^ Regulatory T (Treg) cells, as a critical cell subset for maintaining immune homeostasis and suppressing inflammatory responses, are closely associated with the dysfunction of various autoimmune and inflammatory diseases.^[6]^ In particular, CD39^+^ secreting Treg cells have become a research hotspot in immunomodulation due to their robust immunosuppressive capacity.^[7,8]^ Accumulating evidence has demonstrated that CD39^+^ secreting Treg cells modulate the immune microenvironment and influence the intensity and persistence of inflammatory responses, suggesting their potential involvement in the immunopathological process of IVDD.^[9]^ However, the causal relationship between CD39^+^ secreting Treg cells and IVDD has not been systematically elucidated, which restricts further research and clinical translation in this field.

Metabolic processes play a crucial role in modulating disease susceptibility and unveiling new therapeutic avenues.^[10]^ These small molecules represent the end products of gene-environment interactions, offering potential in identifying new biomarkers and mechanisms of disease.^[10]^ Moreover, potential pathways related to CD39^+^ secreting Treg and IVDD have not been investigated. Gene prediction reveals a significant impact of pimelylcarnitine (C7-DC) on both the CD39^+^ secreting Treg dataset and IVDD. Consequently, C7-DC may serve as a potential mediator between IVDD and CD39^+^ secreting Treg. To minimize the influence of non-genetic confounding factors and reverse causation, the Mendelian randomization (MR) method is adopted.^[11]^ This study through MR aims to investigate the causal relationships, using genetic variations as predictors for specific exposures and their effects on observed outcomes.^[12,13]^ Since an individual’s genetic makeup is fixed at conception, leveraging these variations as instrumental variables (IVs) enhances the credibility of our findings. Moreover, based on the availability of publicly genetic data, this method can be widely applied. The present study therefore aims to determine whether CD39^+^ secreting Treg is causally associated with IVDD and evaluate the mediating effect of C7-DC on the relationship between CD39^+^ secreting Treg and IVDD.

## 2. Methods

### 2.1. Ethics approval

This study was exempt from ethical approval by the Institutional Review Board of Fenghua Hospital of Traditional Chinese Medicine (Ningbo, Zhejiang Province, China). The exemption was granted due to the exclusive use of publicly available, de-identified genome-wide association study (GWAS) summary datasets for secondary data analysis, with no access to individual-level clinical, genetic, or demographic information of research participants. All original datasets utilized in this study had been ethically approved by their respective institutional review boards and were released for open scientific research, with participant privacy fully protected through de-identification and anonymization. This study involved no human subjects recruitment, sample collection, or experimental interventions, and all data analysis strictly followed the data access and usage guidelines of the original dataset providers. Informed consent was not applicable given the nature of secondary analysis of publicly available anonymized data.

### 2.2. Study design

**Figure 1.**
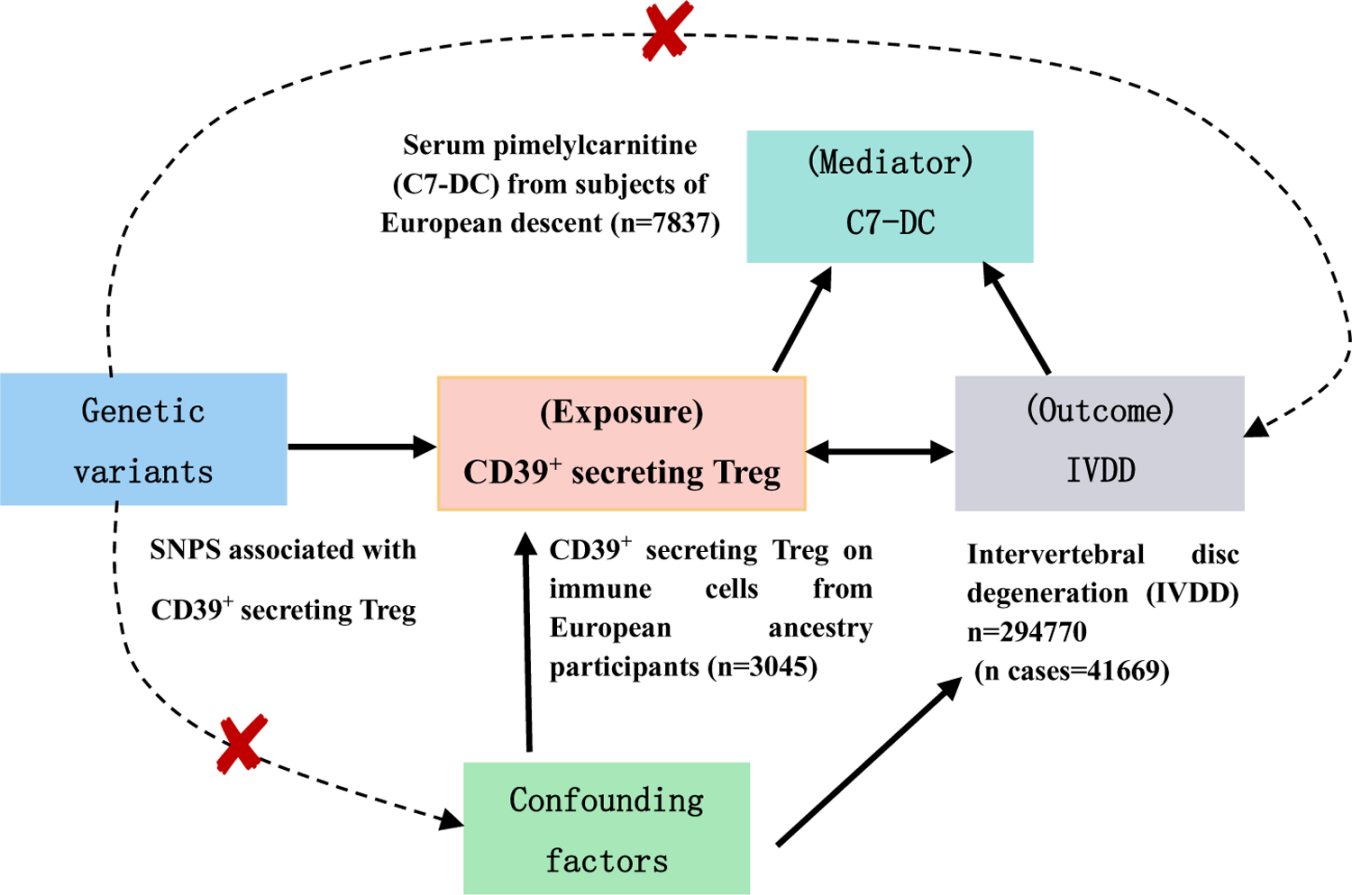
The design of bidirectional Mendelian randomization (MR) study. The “✘” means that genetic variants are not associated with confounders or cannot be directly involved in outcome but via the exposure pathway. Solid paths are significant; dashed paths should not exist in the MR study. SNP = single nucleotide polymorphism.

A two-sample, bidirectional MR approach was adopted to investigate the reciprocal causal relationship between CD39^+^ secreting Treg and IVDD, with the MR study design overviewed in Figure 1. All study findings are detailed in the main text and Supplementary Materials. For genetic variants to be considered as IVs, they must satisfy 3 rigorous criteria^[14]^: a strong association with the exposure of interest, no linkage with potential confounding factors including body mass index, gender, and age, and exclusive influence on the outcome through the exposure rather than direct effects. Summary statistic datasets from recent meta-analyses of GWAS for C7-DC, CD39^+^ secreting Treg, and IVDD were utilized for subsequent analyses. Summary statistic datasets from recent meta-analyses of GWAS for C7-DC, CD39^+^ secreting Treg, and IVDD were utilized.

### 2.3. GWAS summary data sources

Summary statistics for the analysis of CD39^+^ secreting Treg cells were obtained from a comprehensive GWAS by the Blood Cell Consortium focusing on blood cell-related traits, with 3045 participants of European descent included^[15]^ (available at: http://ftp.ebi.ac.uk/pub/databases/gwas/summary_statistics/GCST90001001-GCST90002000/GCST90001495/). IVDD data were retrieved from the FinnGen consortium, which included 41,669 cases and 294,770 controls.^[16]^ Diagnoses of IVDD were established based on the International Classification of Diseases (ICD) codes: ICD-10 M51, ICD-9 722, and ICD-8 275. Further details on the IVDD outcomes can be found in Table S1. IVs for examining metabolites were derived from summary data of a GWAS on the human metabolome, which was carried out among individuals of European ancestry^[17]^ (N = 7837) (accessible at: http://ftp.ebi.ac.uk/pub/databases/gwas/summary_statistics/GCST90200001-GCST90201000/GCST90200101/).

### 2.4. Instrumental variable selection and data harmonization

Single nucleotide polymorphisms (SNPs) achieving genome-wide significance (*P* < 5 × 10^−8^) were selected as IVs. In the absence of SNPs meeting this threshold, variants with *P*-values (*P* < 5 × 10^−5^) were considered as potential IVs. Then, these SNPs were clustered based on linkage disequilibrium (window size = 10,000 kb and *r*^2^ < 0.001). Estimated levels of linkage disequilibrium from the 1000 Genomes Project based on European samples.^[18]^ For exposure-related SNPs absent from the outcome dataset, proxy SNPs were identified via LD tagging. SNPs that were palindromic or could not be unambiguously assigned were not included in the set of IVs for conducting MR studies.^[19]^ To evaluate instrumental variable strength, the F statistic was calculated based on the proportion of variance in exposure explained by the selected SNPs, using the formula: [(*N*−*K*−1)/*K*]/ [*R*^2^/(1–*R*^2^)], where *K* denotes the number of genetic variants and *N* represents the total sample size. Weak IVs (*F*-statistic < 10) were subsequently excluded.^[20]^

### 2.5. MR analysis

**Figure 2.**
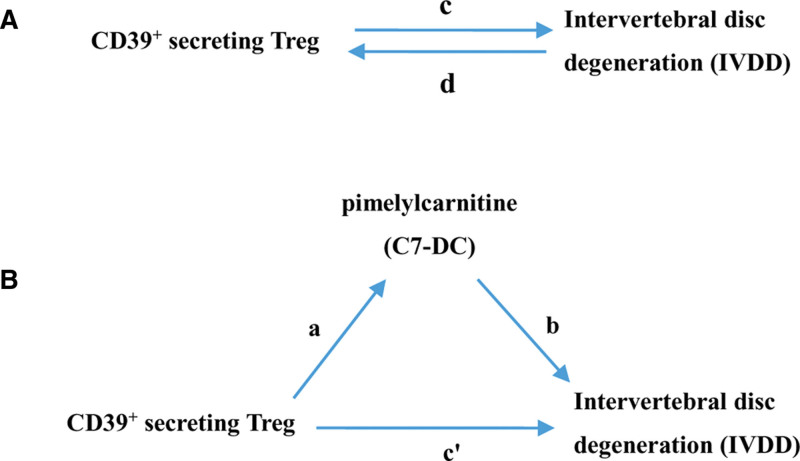
Diagrams illustrating associations examined in this study. (A) The total effect between CD39^+^ secreting Treg and intervertebral disc degeneration (IVDD). c is the total effect using genetically predicted CD39^+^ secreting Treg as exposure and IVDD as outcome. d is the total effect using genetically predicted IVDD as exposure and CD39^+^ secreting Treg as outcome. (B) The total effect was decomposed into: (i) indirect effect using a two-step approach (where a is the total effect of CD39^+^ secreting Treg on pimelylcarnitine (C7-DC), and b is the effect of C7-DC on IVDD) and the product method (a × b) and (ii) direct effect (c′ = c—a × b). Proportion mediated was the indirect effect divided by the total effect.

Figure 2 provides a diagrammatic summary of the analytical framework. A bidirectional, two-sample MR design was implemented to examine the reciprocal causal relationship between CD39^+^ secreting Treg and IVDD (Fig. 2A), defined as the total effect.

In this study, TwoSampleMR package with R software (version 4.2.3) was used for MR analyses.^[21]^ The inverse-variance-weighted (IVW) method was applied as the primary analytical approach, which estimates causal effects of genetically predicted exposure on the outcome via weighted regression of SNP-specific Wald ratios (i.e., beta outcome/beta exposure). Several sensitivity analyses were conducted, including the weighted median (WM) method and the MR-Egger regression method.^[22]^ Among them, the WM method selects the median estimate to compute the causal effect.^[23]^ MR-Egger regression method effectively tests the null causal hypothesis and gives a consistent estimate to causality, even if no genetic variants are valid IVs.^[24]^ The MR-Egger regression method is robust to horizontal pleiotropy. Heterogeneity was quantified using Cochran *Q* statistics and *I*^2^ statistics^.[25]^ Larger *I*^2^ values show increasing heterogeneity. Besides, “leave-one-out analysis” by removal of every single SNP at turn could make sure the reliability of the results.

### 2.6. Mediation analysis

A mediation analysis was further performed using a two-step MR design to investigate whether C7-DC mediates the causal pathway from CD39^+^ secreting Treg to IVDD (Fig. 2B). The overall effect can be decomposed into an indirect effect (through mediators) and a direct effect (without mediators) effect.^[26]^ The total effect of CD39^+^ secreting Treg on IVDD was decomposed into 1) direct effects of CD39^+^ secreting Treg on IVDD (c’ in Fig. 2B) and 2) indirect effects mediated by CD39^+^ secreting Treg through the mediator (a × b in Fig. 2B). The mediated percentage was determined by dividing the indirect effect by the total effect. Corresponding 95% CIs were derived using the delta method.^[27]^

## 3. Results

### 3.1. Association of CD39^+^ secreting Treg with IVDD

One SNP could not be matched with any proxy SNPs using the LDlink online tool (https://ldlink.nih.gov/?tab=ldproxy) and was subsequently excluded from our analysis. The Phenoscanner database was used to investigate associations between the remaining CD39^+^ secreting Treg-related SNPs and established IVDD risk factors, including body mass index, body fat percentage, and bone mineral density. This search yielded no SNPs associated with these traditional risk factors. Ultimately, 26 LD-independent genetic variants were taken as IVs for CD39^+^ secreting Treg (Fig. 2, Table S2).

**Figure 3.**
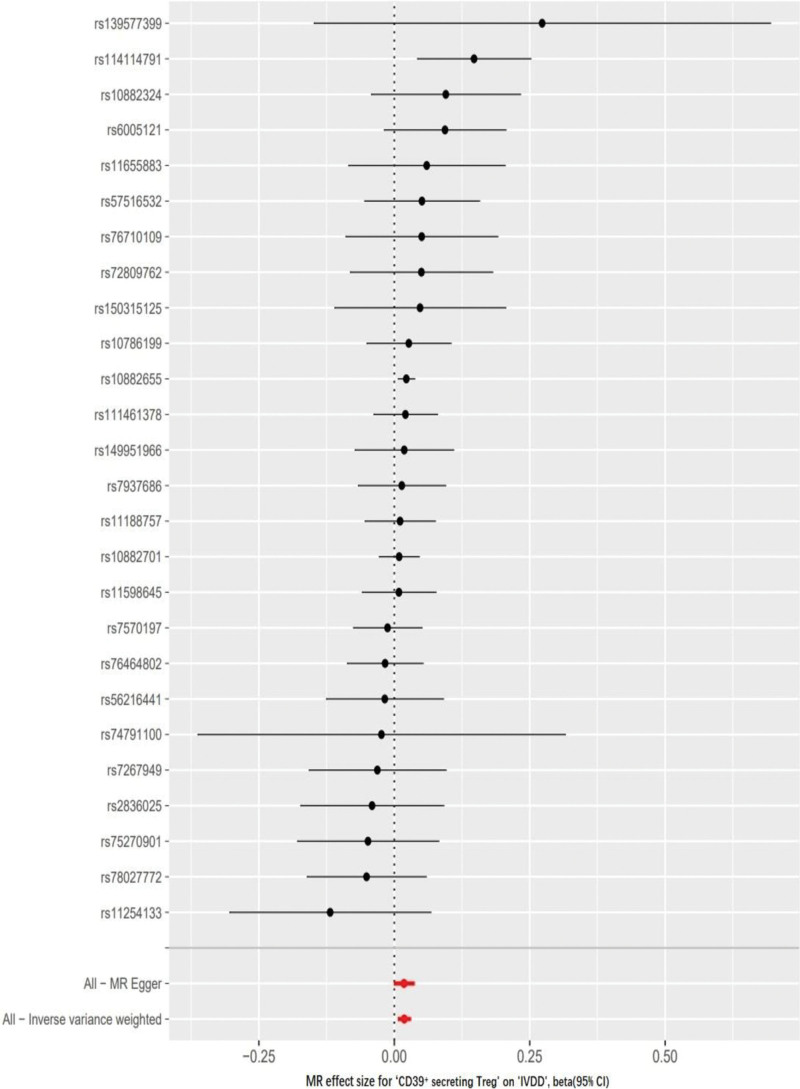
Forest plot of the causal effects of CD39^+^ secreting Treg associated SNPs on intervertebral disc degeneration (IVDD). Data are expressed as beta values with 95% CI. CI = confidence interval, SNP = single nucleotide polymorphism.

**Figure 4.**
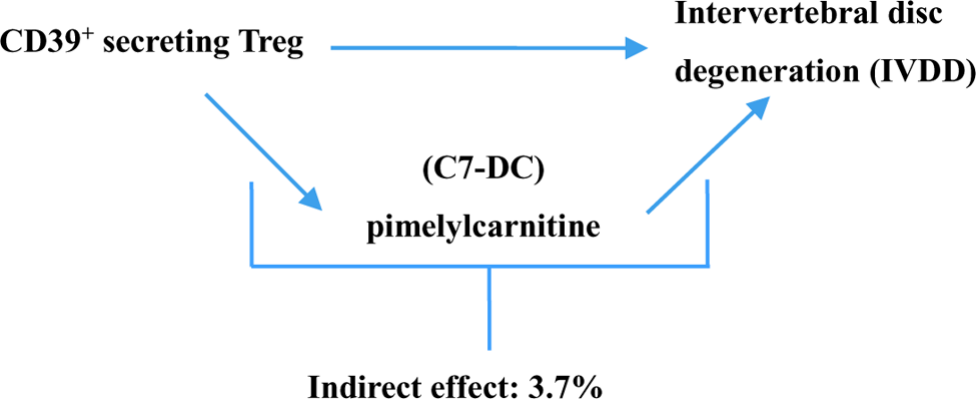
Schematic diagram of the pimelylcarnitine (C7-DC) mediation effect.

Multiple MR approaches, including MR-Egger, WM regression, IVW and weighted mode analysis, were applied to investigate the causal effect of genetically predicted CD39^+^ secreting Treg on IVDD (Figs. 3 and 4). Across all MR methods, there was broad and consistent support for the positive association of CD39^+^ secreting Treg with IVDD (WM regression odds ratio (OR) per SD increase in IVDD = 1.022 [95% confidence interval [CI]: 1.007–1.036], *P* < .01; IVW OR per SD increase in IVDD = 1.019 [95% CI: 1.007–1.031], *P* < .01; Weighted mode OR per SD increase in IVDD = 1.019 [95% CI: 1.00–1.03], *P* < .05). As shown in Table 1, heterogeneity wasn’t detected by Cochran *Q* test (*P* = .727). And MR-Egger analysis did not suggest any directional pleiotropy for the IVs (*P* = .948). No reverse causal association was identified between genetically predicted IVDD and CD39^+^ secreting Treg (i.e., no causality for genetically predicted IVDD on CD39^+^ secreting Treg). The OR was 0.909 [95% CI: 0.754–1.096; *P* = .48] by using the IVW method.

**Table 1:** MR results of the causal effects of C7-DC with CD39^+^ secreting Treg and IVDD.

Exposures	Outcomes	No. of SNPS	Method	OR (95% CI)	*P*	Heterogeneity test		Pleiotropy test
						Cochran *Q (I*^2^)	*P* *	*P* intercept
CD39^+^ secreting Treg	IVDD	26	MR Egger	1.018 (0.999, 1.027)	.073	20.38 (0.0%)	.727	.948
		26	(WM) regression	1.022 (1.007, 1.036)	.003			
		26	IVW	1.019 (1.007, 1.031)	.002			
		26	WM	1.019 (1.005, 1.034)	.014			
IVDD	CD39^+^ secreting Treg	37	MR Egger	0.584 (0.230, 1.489)	.268	35.77 (0.0%)	.480	.351
		37	WM	0.780 (0.593, 1.026)	.076			
		37	IVW	0.909 (0.754, 1.096)	.320			
CD39^+^ secreting Treg	C7-DC	25	MR Egger	0.959 (0.922, 0.998)	.053	23.51 (0.0%)	.490	.459
		25	IVW	0.971 (0.948, 0.995)	.018			
C7-DC	IVDD	28	MR Egger	0.888 (0.828, 0.952)	.003	32.99 (18%)	.197	.059
		28	IVW	0.944 (0.911, 0.978)	.002			

C7-DC = pimelylcarnitine, CI = confidence interval, IVDD = intervertebral disc degeneration, IVW = inverse variance weighted, MR = Mendelian randomization, OR = odds ratio, SNPs = single nucleotide polymorphisms, WM = weighted median.

* *P* represents heterogeneity.

### 3.2. Association of CD39^+^ secreting Treg with C7-DC

A total of 25 genome-wide significant SNPs were extracted as IVs following the exclusion of palindromic, ambiguous SNPs and variants without proxy SNPs (Table S3). The variance explained by and Fstatistic for SNPs instrumenting CD39^+^ secreting Treg exposure were 2.98% and 155.73, respectively. According to the IVW, and MR–Egger, genetically predicted CD39^+^ secreting Treg was found to be negatively associated with C7-DC levels (MR-Egger method, OR, 0.959; [95% CI: 0.922–0.988], *P* = .053; IVW method, OR, 0.971; [95% CI: 0.948–0.995], *P* < .05). The results are shown in Table 1. No heterogeneity was detected by Cochran *Q* test for total IVDD (*P* = .49). These findings were similar across other MR estimates. MR-Egger analysis did not suggest any directional pleiotropy for the IVs (*P* = .459). The results are shown in Table 1.

### 3.3. Association of C7-DC with IVDD

Genetic instruments for C7-DC explained 0.3% of its variance, with an *F*-statistic of 24.3. As shown in Table S4, we presented all genetic instruments associated with C7-DC at the genome-wide significance level (*P* < 5 × 10^−5^). As shown in Table 1, genetically predicted C7-DC was significantly negatively correlated with IVDD [OR = 0.944, 95% CI: 0.911–0.978; *P* = .002] by using the IVW method. The estimation direction of MR-Egger method was consistent [OR = 0.888, 95% CI: 0.828–0.952; *P* = .003]. Similar findings were obtained using other MR estimation methods. No heterogeneity was detected by Cochran *Q* test (*P* = .197). MR-Egger analysis did not suggest any directional pleiotropy for the IVs (*P* = .059).

### 3.4. Proportion of the association between CD39^+^ secreting Treg and IVDD mediated by C7-DC

C7-DC was analyzed as a mediator of the pathway from CD39^+^ secreting Treg to IVDD. CD39^+^ secreting Treg was found to be associated with decreased C7-DC, which in turn was associated with an increased risk of IVDD. As shown in Figure 4, C7-DC accounted for 9.1% of the increased risk of IVDD associated with CD39^+^ secreting Treg.

Scatter plots, funnel plots and leave-one-out sensitivity analysis are presented in Figs. S1 S3.

## 4. Discussion

IVDD is mainly characterized by a reduction in disc height, decreased hydration, and a diminished capacity to absorb pressure. The pathogenesis of IVDD largely involves the activation of the body’s autoimmune response. The underlying mechanisms of IVDD are heavily influenced by the initiation of the autoimmune response within the body. In cases of IVDD, the fibrous ring encircling the degenerating disc tissue may tear, causing the nucleus pulposus to protrude. A pivotal event in IVDD progression is the rupture of the fibrous annulus that encases the deteriorating disc, causing the inner nucleus pulposus to bulge outward. Such bulging incites a potent inflammatory reaction in the nearby cellular environment. Additionally, the progression of IVDD is believed to be linked to the activation of immune cells, including macrophages and T cells within the intervertebral disc.^[28,29]^ The results of the two-sample MR analysis demonstrated a positive association between genetically predicted CD39^+^ secreting Treg and the risk of IVDD, The related research indicates that immune infiltration analysis demonstrated that the Treg cells had significant infiltration, confirming our conclusion.^[30]^ The activation of inflammatory factors stimulates the body’s own immune response, thereby promoting the progression of IVDD. A higher expression of CD39^+^ secreting Treg is associated with a more pronounced IVDD. However, there was no strong evidence supporting the reverse causality, suggesting that genetically predicted IVDD had no significant effect on CD39^+^ secreting Treg.

The potential mechanism of action of secreting Treg cells may involve immunomodulation, wherein they generate and express repair factors that act on immune targets, thus coordinating the promotion of tissue repair by parenchymal cells following injury.^[31]^ For instance, Tregs are known to directly induce anti-inflammatory macrophages, playing a crucial role in repair during the initiation, maintenance and resolution phases.^[32]^ They are significant producers of IL-10, a cytokine that directly influences fibroblasts.^[33]^ Furthermore, TGF-β1, secreted by Treg cells, is pivotal for their differentiation and it plays a role in reinforcing the development of dense fibrous tissues at sites of wound healing.^[34]^ Within the immune system, extracellular ATP acts as a “natural adjuvant,” manifesting a range of proinflammatory responses. It is released by damaged cells as an indicator of trauma and cell death but can be inactivated by CD39, an ectoenzyme proficient in converting ATP into AMP.^[35]^ CD39 is expressed primarily by immunoregulatory Treg cells.^[36]^ While the effects of extracellular ATP on the immune system are widely recognized,^[37]^ the role of CD39 in immune modulation remains to be fully elucidated. Besides removing a proinflammatory stimulus, it may also act in concert with CD73, another ectonucleotidase present on the surface of lymphocytes, to produce adenosine.^[38]^ This nucleoside exhibits mostly inhibitory and antiproliferative effects,^[39]^ so the overall effect of CD39 activity should be mainly immune suppressive. Based on these insights, we propose that intervertebral disc-infiltrating CD39^+^Treg have a critical impact on IVDD.

C7-DC is a medium-chain AC acylcarnitine. The primary function of acylcarnitines involves the translocation of acyl groups, which include organic acids and fatty acids, from the cytoplasm into mitochondria, facilitating their breakdown through beta-oxidation to generate energy.^[40]^ Acylcarnitines are important markers in metabolic studies of many diseases, including metabolic, cardiovascular, and neurological disorders.^[41]^ The current research reports on the effects of C7-DC on human physiological functions are relatively limited. Recent findings from a study analyzing ante mortem blood and post mortem brain specimens within 2 community-oriented longitudinal cohorts focusing on aging and dementia have shown that C7-DC is a significant predictor of reduced Alzheimer disease risk over a follow-up period of 4.5 years, regardless of age, gender, and educational background.^[42]^ Another study indicates that patients with Early-Stage Alzheimer disease have lower levels of C7-DC in their serum.^[43]^ Previous studies have shown that individuals suffering from Heart failure with reduced ejection fraction,^[44]^ early stage hepatocellular carcinoma,^[45]^ and psoriasis^[46]^ have lower serum concentrations of C7-DC. However, a study reported serum C7-DC as an adverse factor affecting human health, presenting a contrasting result that indicated serum C7-DC may exacerbate the development and progression of chronic or end-stage kidney disease.^[47]^ Currently, there are no reports in the literature regarding the correlation between serum C7-DC and IVDD. The MR analysis indicated a negative association between genetically predicted CD39^+^ secreting Treg and C7-DC levels. Additionally, C7-DC was found to be negatively correlated with IVDD. Mediation analysis revealed that C7-DC accounted for 9.1% of the increased risk of IVDD associated with CD39^+^ secreting Treg.

From a biomechanical and anatomical perspective, the interplay between CD39^+^ secreting Treg cells, C7-DC, and IVDD provides critical insights for clinical practitioners. The lumbosacral junction, a key anatomical region prone to mechanical stress concentration, is frequently involved in IVDD pathogenesis. Lumbosacral transitional vertebra (LSTV), a common congenital anatomical variant, alters the normal spinal biomechanics by disrupting the lumbosacral alignment and reducing mobility between the transitional vertebra and the sacrum.^[48]^ This anatomical anomaly leads to asymmetric force distribution and abnormal torque moments on adjacent segments, particularly the intervertebral disc immediately cephalad to the transitional level.^[49]^ Such altered biomechanics increase the mechanical burden on the disc, accelerating structural damage such as reduced disc height, annulus fibrosus fissures, and nucleus pulposus dehydration – core pathological features of IVDD.^[50]^ Clinically, patients with LSTV, especially type III LSTV characterized by complete bony fusion between the transverse process and sacral ala, exhibit higher rates of disc protrusion and vertebral slippage.^[51]^ This underscores the need for clinicians to integrate anatomical assessments (e.g., identification of LSTV on radiographs) with biomechanical considerations when evaluating IVDD risk, as structural variants directly modulate the mechanical environment in which CD39^+^ Treg cells and C7-DC exert their effects.

Skeletal muscle, as a key anatomical component maintaining spinal stability, exerts a profound biomechanical influence on IVDD progression.^[52]^ Appendicular lean mass acts as a protective factor against IVDD primarily by enhancing the load-bearing capacity of the spine and reducing mechanical stress on intervertebral discs.^[53]^ From an anatomical standpoint, paraspinal muscles (e.g., multifidus, erector spinae) and appendicular muscles work synergistically to stabilize the lumbar spine during dynamic movements.^[52]^ Reduced appendicular lean mass, a hallmark of sarcopenia, compromises this stabilizing function, leading to increased segmental mobility and repetitive microtrauma to the intervertebral disc and vertebral endplates.^[54]^ This mechanical damage triggers immune responses, including the infiltration of CD39^+^ Treg cells, which may further modulate the local inflammatory microenvironment.^[9]^ Conversely, excessive hand grip strength, identified as a risk factor for IVDD,^[53]^ may indirectly reflect abnormal muscle force transmission to the spine. Anatomically, the psoas major and other hip flexors connect the appendicular skeleton to the lumbar spine; excessive contraction of these muscles can generate increased compressive and shear forces on the lumbar discs, exacerbating disc degeneration.^[55]^ Clinicians should therefore recognize that muscle-related biomechanical factors interact with the immunometabolic pathway identified in this study. For example, optimizing muscle mass and function through targeted rehabilitation may mitigate the mechanical stress that potentiates the pro-degenerative effects of CD39^+^ Treg cell infiltration.

This study has several limitations. First, we selected one immune cell type most relevant to IVDD for analysis. It’s accurate that disc degeneration isn’t driven by a single type of immune cell. Instead, it involves a complex network of immune reactions. Therefore, conducting a comprehensive study of various immune cells could provide a more in-depth understanding of the pathophysiology of IVDD. Second, since C7-DC mediates only a fraction of the effect, it is essential to identify and quantify other potential mediators in the pathway between CD39^+^ secreting Treg and IVDD. Furthermore, considering that inflammatory responses are another important factor affecting IVDD, treating inflammatory factors as mediating factors could provide valuable information regarding the influences on disc degeneration. Third, Instrumental Variable Constraints: The MR analysis is inherently dependent on the validity of the chosen IVs. While we adhered to stringent selection criteria, the potential for pleiotropic effects cannot be completely excluded. This limitation underscores the need for cautious interpretation of the causality inferred from our analysis. Fourth, we used summary-level statistics in our study, not individual-level data. Therefore, we cannot further explore causal links between subgroups such as females and males.

## 5. Conclusion

In conclusion, this study identifies a causal relationship between CD39^+^ secreting Tregs and IVDD. While C7-DC mediates a small proportion of this effect, the majority of the mechanism by which CD39^+^ secreting Tregs influence IVDD remains unclear. Further research is therefore required to investigate additional risk factors as potential mediators.

## Acknowledgments

The authors thank all the participants and researchers for their participation in this MR study. All authors interpreted the data, critically revised the manuscript for important intellectual content, approved the final version of the manuscript, and agreed to be responsible for all aspects of the work. The corresponding author attests that all listed coauthors meet authorship criteria and that no others meeting the criteria have been omitted.

## Author contributions

**Conceptualization:** Na Li.

**Data curation:** Na Li, Cimin Shen, Zhe-Nv Huang, Dan-Yun Hua.

**Formal analysis:** Na Li, Zhuo-Yi Shao.

**Funding acquisition:** Cimin Shen.

**Investigation:** Na Li, Jie-Tao Wu.

**Methodology:** Cimin Shen, Jie-Tao Wu, Dan-Yun Hua.

**Project administration:** Na Li, Cimin Shen, Jie-Tao Wu.

**Resources:** Na Li, Cimin Shen, Zhe-Nv Huang, Lu Wang.

**Software:** Na Li, Lu Wang.

**Supervision:** Zhe-Nv Huang.

**Validation:** Na Li, Zhuo-Yi Shao.

**Visualization:** Zhuo-Yi Shao, Dan-Yun Hua.

**Writing – original draft:** Na Li, Cimin Shen, Zhe-Nv Huang, Zhuo-Yi Shao, Jie-Tao Wu, Dan-Yun Hua, Lu Wang.

**Writing – review & editing:** Na Li, Cimin Shen, Zhe-Nv Huang, Zhuo-Yi Shao, Jie-Tao Wu, Dan-Yun Hua, Lu Wang.














