# Supplementary material for: Genetically predicted serum Pimelylcarnitine mediates the association between CD39+ secreting Treg cells and intervertebral disc degeneration
Source: Medicine (Baltimore). 2026 May 15;105(20):e48540. doi: 10.1097/MD.0000000000048540 (PMC13183168; doi:10.1097/MD.0000000000048540)
Supplement: Supplementary file 1 [file medi-105-e48540-s001.docx]

| Supplementary Table S1 Baseline characteristics of the study population | | | | | | | | | |
| --- | --- | --- | --- | --- | --- | --- | --- | --- | --- |
| **Traits** | **Consortium** | **ID** | **Sample Size** | **N cases** | **N controls** | **Total number of SNPs tested** | **Definition/inclusion criteria** | **Population Studied** |  |
| CD39^+^ secreting Treg | Blood Cell Consortium (BCX) | GCST90001495 | 3405 | NA | NA | 1048575 | NA | European (Italy) |  |
| Pimeloylcarnitine  **（C7-DC）** | BCX | GCST90200101 | 7387 | NA | NA | 972845 | NA | European (Canada) |  |
| IVDD | Finngen (R10) | M13_INTERVERTEB | 336439 | 41669 | 294770 | 21304571 | CD-10M51, ICD-9 722, ICD-8 725; excluded ICD-9 7220\|7224\|7227\|7228Av, ICD-87250 | European |  |

# Supplementary Tables
