# Supplementary material for: Genetically predicted serum Pimelylcarnitine mediates the association between CD39+ secreting Treg cells and intervertebral disc degeneration
Source: Medicine (Baltimore). 2026 May 15;105(20):e48540. doi: 10.1097/MD.0000000000048540 (PMC13183168; doi:10.1097/MD.0000000000048540)
Supplement: Supplementary file 3 [file medi-105-e48540-s003.docx]

| Supplementary Table S3 Characteristics of SNPs used as genetic instruments for CD39^+^ secreting Treg on **C7-DC** in the present MR study (PMID:36635386 for **C7-DC**) | | | | | | | | | | | | | | |
| --- | --- | --- | --- | --- | --- | --- | --- | --- | --- | --- | --- | --- | --- | --- |
| Exposure | SNP | Chr | Position | Nearest gene | EA | NEA | EAF | SNP-Exposure association | | | *R^2^* ^a^ | *F*-statistic ^b^ | Proxy ^d^ | Confounder ^e^ |
|  |  |  |  |  |  |  |  | Beta | SE | *P* value |  |  |  |  |
| CD39^+^ secreting Treg | rs10786199 | 10 | 97069472 | SORBS1 | A | G | 0.2894 | -0.1932 | 0.02906 | 3.39E-11 | 0.012814588 | 44.17411496 |  |  |
| CD39^+^ secreting Treg | rs10882655 | 10 | 97474371 | ENTPD1 | A | G | 0.532 | -0.9594 | 0.02046 | 1.00E-200 | 0.392377942 | 2197.52084 |  |  |
| CD39^+^ secreting Treg | rs10882701 | 10 | 97741071 | CC2D2B | C | A | 0.514 | -0.4069 | 0.02528 | 3.19E-56 | 0.07070615 | 258.9202832 |  |  |
| CD39^+^ secreting Treg | rs111461378 | 20 | 15575550 | MACROD2 | C | T | 0.0028 | -1.066 | 0.2404 | 9.47E-06 | 0.00574153 | 19.6512561 |  |  |
| CD39^+^ secreting Treg | rs11188757 | 10 | 98205222 | TLL2 | C | A | 0.2213 | -0.2549 | 0.03174 | 1.33E-15 | 0.018589151 | 64.45708468 |  |  |
| CD39^+^ secreting Treg | rs11254133 | 10 | 16697829 | RSU1 | T | C | 0.1317 | -0.1723 | 0.03826 | 6.88E-06 | 0.005920856 | 20.26867954 |  |  |
| CD39^+^ secreting Treg | rs114114791 | 1 | 25840181 | TMEM57 | G | A | 0.0304 | 0.3538 | 0.07416 | 1.91E-06 | 0.006639965 | 22.74683869 |  |  |
| CD39^+^ secreting Treg | rs11598645 | 10 | 68351521 | CTNNA3 | C | T | 0.011 | -0.6337 | 0.1381 | 4.64E-06 | 0.006145901 | 21.0438351 |  |  |
| CD39^+^ secreting Treg | rs11655883 | 17 | 75234757 | SEC14L1 | G | A | 0.2902 | 0.1294 | 0.02794 | 3.74E-06 | 0.006259958 | 21.43683089 |  |  |
| CD39^+^ secreting Treg | rs139577399 | 7 | 23203415 | KLHL7 | C | T | 0.0414 | -0.2948 | 0.06509 | 6.14E-06 | 0.005988262 | 20.50082074 |  |  |
| CD39^+^ secreting Treg | rs149951966 | 17 | 41137136 | RUNDC1 | C | T | 0.1035 | 0.2069 | 0.04388 | 2.51E-06 | 0.006487004 | 22.21941274 |  |  |
| CD39^+^ secreting Treg | rs150315125 | 8 | 10090715 | MSRA | T | A | 0.0295 | -0.4106 | 0.08147 | 4.89E-07 | 0.007404532 | 25.38559168 |  |  |
| CD39^+^ secreting Treg | rs2836025 | 21 | 39232216 | KCNJ | T | C | 0.0762 | -0.2143 | 0.04774 | 7.40E-06 | 0.005883014 | 20.13837138 |  |  |
| CD39^+^ secreting Treg | rs56216441 | 18 | 65545793 | DSEL | T | A | 0.1373 | -0.1636 | 0.03693 | 9.74E-06 | 0.005730532 | 19.61339687 |  |  |
| CD39^+^ secreting Treg | rs57516532 | 7 | 1172869 | C7orf50 | T | C | 0.0342 | 0.3162 | 0.07091 | 8.48E-06 | 0.005805811 | 19.87254977 |  |  |
| CD39^+^ secreting Treg | rs6005121 | 22 | 27065117 | CRYBA4 | G | A | 0.8974 | -0.2011 | 0.04185 | 1.62E-06 | 0.006735672 | 23.07693089 |  |  |
| CD39^+^ secreting Treg | rs7267949 | 20 | 17586584 | DSTN | T | C | 0.0057 | -0.7497 | 0.1688 | 9.25E-06 | 0.00575976 | 19.71400988 |  |  |
| CD39^+^ secreting Treg | rs72809762 | 10 | 97294154 | SORBS1 | T | C | 0.0675 | 0.4322 | 0.05126 | 4.98E-17 | 0.020451315 | 71.04886659 |  |  |
| CD39^+^ secreting Treg | rs74791100 | 9 | 86052403 | FRMD3 | C | T | 0.0123 | 0.5538 | 0.1185 | 3.09E-06 | 0.00637346 | 21.82800452 |  |  |
| CD39^+^ secreting Treg | rs75270901 | 7 | 80556281 | SEMA3C | A | G | 0.0512 | 0.2585 | 0.05807 | 8.78E-06 | 0.005786023 | 19.80442673 |  |  |
| CD39^+^ secreting Treg | rs7570197 | 2 | 79140712 | REG3G | C | T | 0.012 | 0.5826 | 0.1287 | 6.23E-06 | 0.0059822 | 20.47994093 |  |  |
| CD39^+^ secreting Treg | rs76464802 | 7 | 67769786 | TYW1 | A | G | 0.015 | -0.4852 | 0.1056 | 4.47E-06 | 0.006161867 | 21.0988401 |  |  |
| CD39^+^ secreting Treg | rs76710109 | 10 | 131817745 | LINC00959 | C | T | 0.1671 | -0.1594 | 0.03494 | 5.24E-06 | 0.006075293 | 20.80059057 |  |  |
| CD39^+^ secreting Treg | rs78027772 | 7 | 126248228 | GRM8 | A | G | 0.0846 | 0.2257 | 0.04708 | 1.71E-06 | 0.006704273 | 22.9686274 |  |  |
| CD39^+^ secreting Treg | rs7937686 | 11 | 130313067 | ADAMTS15 | G | C | 0.9091 | 0.1946 | 0.04346 | 7.81E-06 | 0.00585382 | 20.03784721 |  |  |
| Abbreviation: SNP, single nucleotide polymorphism; Chr, chromosome; EA, Effect allele; NEA, Non-effect allele; EAF, effect allele frequency; SE, standard error,.  ^a^ *R^2^* was calculated using the following formula: (2×EAF×(1-EAF)×beta^2^)/[(2×EAF×(1-EAF)×beta^2^)+(2×EAF×(1-EAF)×N×SE^2^)], where EAF is the effect allele frequency, beta is the estimated effect on CD39^+^ secreting Treg, Ν is the sample size of the GWAS for the SNP-CD39^+^ secreting Treg association and SE is the standard error of the estimated effect.  ^b^ *F* statistic was calculated using the following formula: *R^2^*(N-2)/(1-*R^2^*), where *R^2^* is the proportion of variance in CD39^+^ secreting Treg explained by each instrument and N is the sample size of the GWAS for the SNP-urate or gout association.  ^d^ Proxy SNPs not available on the online platform **LDlink** (**https://ldlink.nih.gov/?tab=ldproxy**/) were removed.  ^e^ SNPs associated with confounding factors were removed after searching Phenoscanner database. | | | | | | | | | | | | | | |
