# Supplementary material for: Genetically predicted serum Pimelylcarnitine mediates the association between CD39+ secreting Treg cells and intervertebral disc degeneration
Source: Medicine (Baltimore). 2026 May 15;105(20):e48540. doi: 10.1097/MD.0000000000048540 (PMC13183168; doi:10.1097/MD.0000000000048540)
Supplement: Supplementary file 4 [file medi-105-e48540-s004.docx]

| Supplementary Table S4 Characteristics of SNPs used as genetic instruments for **C7-DC** on IVDD in the present MR study | | | | | | | | | | | | | | | |
| --- | --- | --- | --- | --- | --- | --- | --- | --- | --- | --- | --- | --- | --- | --- | --- |
| Exposure | SNP | Chr | Position | Nearest gene | EA | NEA | EAF | SNP-Exposure association | | | | *R^2^* ^a^ | *F*-statistic ^b^ | Proxy ^d^ | Confounder ^e^ |
|  |  |  |  |  |  |  |  | Beta | SE | | *P* value |  |  |  |  |
| C7-DC | rs10148392 | 14 | 100559962 | BEGAIN | G | A | 0.236753 | -0.0823651 | | 0.018451 | 8.05E-06 | 0.002451755 | 19.25671241 |  |  |
| C7-DC | rs11753995 | 6 | 160154334 | SLC22A1 | A | G | 0.167542 | 0.207632 | | 0.0205192 | 4.55E-24 | 0.01202554 | 95.36694707 |  |  |
| C7-DC | rs12132488 | 1 | 46923941 | CYP4A11 | C | T | 0.875081 | 0.201202 | | 0.0235427 | 1.27E-17 | 0.008850572 | 69.96344757 |  |  |
| C7-DC | rs12208357 | 6 | 160122116 | SLC22A1 | T | C | 0.0708687 | 0.313369 | | 0.0296991 | 5.00E-26 | 0.012932236 | 102.6515822 |  |  |
| C7-DC | rs12998050 | 2 | 153646797 | RPRM | T | C | 0.0382912 | 0.197639 | | 0.0404273 | 1.01E-06 | 0.002876854 | 22.60518478 |  |  |
| C7-DC | rs13355396 | 5 | 3617313 | IRX1 | G | A | 0.0497084 | -0.159825 | | 0.0355355 | 6.87E-06 | 0.002413271 | 18.95371886 |  |  |
| C7-DC | rs138321943 | 1 | 242745717 | PLD5 | G | A | 0.00682638 | 0.432293 | | 0.0943261 | 4.58E-06 | 0.002533973 | 19.90411746 |  |  |
| C7-DC | rs139300931 | 6 | 160206065 | SLC22A2 | T | A | 0.0143464 | 0.324551 | | 0.0652183 | 6.48E-07 | 0.00297895 | 23.40980628 |  |  |
| C7-DC | rs143969294 | 2 | 153646797 | RPRM | A | G | 0.0314408 | -0.235035 | | 0.0445852 | 1.35E-07 | 0.003364456 | 26.44949975 |  |  |
| C7-DC | rs147722382 | 14 | 43985091 | FSCB | T | C | 0.0114059 | -0.321722 | | 0.0727206 | 9.69E-06 | 0.002334206 | 18.33128914 |  |  |
| C7-DC | rs2152725 | 13 | 20926033 | XPO4 | C | A | 0.63326 | -0.0713909 | | 0.0161291 | 9.59E-06 | 0.002367315 | 18.59192584 |  |  |
| C7-DC | rs2214195 | 16 | 20488075 | ACSM2A | A | C | 0.803057 | -0.0885729 | | 0.0193667 | 4.80E-06 | 0.002481525 | 19.49111565 |  |  |
| C7-DC | rs2999545 | 1 | 151931670 | THEM4 | T | A | 0.608593 | -0.100931 | | 0.015718 | 1.35E-10 | 0.004853273 | 38.21083876 |  |  |
| C7-DC | rs34174649 | 8 | 6318788 | MCPH1 | G | A | 0.623444 | -0.0715181 | | 0.0161237 | 9.18E-06 | 0.002401535 | 18.8613243 |  |  |
| C7-DC | rs4752006 | 10 | 116724584 | HSPA12A | T | C | 0.241605 | -0.0810486 | | 0.0180794 | 7.36E-06 | 0.002407257 | 18.90636895 |  |  |
| C7-DC | rs62140628 | 2 | 50866064 | NRXN1 | C | T | 0.0425723 | 0.171097 | | 0.0384405 | 8.55E-06 | 0.002386426 | 18.74237133 |  |  |
| C7-DC | rs62343480 | 5 | 4419433 | ADAMTS16 | G | T | 0.288294 | -0.077633 | | 0.0170828 | 5.51E-06 | 0.002473198 | 19.42555307 |  |  |
| C7-DC | rs7205868 | 16 | 84366921 | ATP2C2 | T | C | 0.35393 | -0.0775845 | | 0.0165061 | 2.60E-06 | 0.002752814 | 21.62783559 |  |  |
| C7-DC | rs73045003 | 7 | 2612362 | IQCE | T | C | 0.123171 | -0.104728 | | 0.0236849 | 9.79E-06 | 0.002369076 | 18.60578876 |  |  |
| C7-DC | rs7410998 | 1 | 928176 | SAMD11 | A | G | 0.923245 | -0.146656 | | 0.0299431 | 9.69E-07 | 0.003048269 | 23.95621357 |  |  |
| C7-DC | rs74931927 | 14 | 78240921 | NRXN3 | A | T | 0.0082523 | -0.379133 | | 0.0849675 | 8.12E-06 | 0.002352824 | 18.47784855 |  |  |
| C7-DC | rs75646959 | 16 | 81796915 | PLCG2 | T | C | 0.0114377 | 0.316629 | | 0.0713218 | 9.02E-06 | 0.002267118 | 17.80323133 |  |  |
| C7-DC | rs7788658 | 7 | 54684693 | SEC61G | T | C | 0.178604 | 0.0944548 | | 0.0206348 | 4.71E-06 | 0.002617712 | 20.56360131 |  |  |
| C7-DC | rs7824299 | 8 | 82929574 | SNX16 | G | A | 0.407895 | 0.0748937 | | 0.01584 | 2.27E-06 | 0.002709366 | 21.2855531 |  |  |
| C7-DC | rs78290674 | 13 | 113364849 | GRTP1 | C | G | 0.0566341 | 0.162428 | | 0.0333278 | 1.10E-06 | 0.002819097 | 22.15006547 |  |  |
| C7-DC | rs7850579 | 9 | 9571300 | PTPRD | A | G | 0.316741 | -0.0815797 | | 0.0166125 | 9.07E-07 | 0.002880606 | 22.63474913 |  |  |
| C7-DC | rs7905852 | 10 | 13408911 | BEND7 | G | A | 0.276428 | -0.0787176 | | 0.0173404 | 5.64E-06 | 0.002478777 | 19.46947879 |  |  |
| C7-DC | rs8002490 | 13 | 43683887 | ENOX1 | A | G | 0.0308105 | -0.203454 | | 0.0447621 | 5.49E-06 | 0.002472122 | 19.41707762 |  |  |
| Abbreviation: SNP, single nucleotide polymorphism; Chr, chromosome; EA, Effect allele; NEA, Non-effect allele; EAF, effect allele frequency; SE, standard error,.  ^a^ *R^2^* was calculated using the following formula: (2×EAF×(1-EAF)×beta^2^)/[(2×EAF×(1-EAF)×beta^2^)+(2×EAF×(1-EAF)×N×SE^2^)], where EAF is the effect allele frequency, beta is the estimated effect on CD39^+^ secreting Treg, Ν is the sample size of the GWAS for the SNP-CD39^+^ secreting Treg association and SE is the standard error of the estimated effect.  ^b^ *F* statistic was calculated using the following formula: *R^2^*(N-2)/(1-*R^2^*), where *R^2^* is the proportion of variance in **C7-DC** explained by each instrument and N is the sample size of the GWAS for the SNP-urate or gout association.  ^d^ Proxy SNPs not available on the online platform **LDlink** (**https://ldlink.nih.gov/?tab=ldproxy**/) were removed.  ^e^ SNPs associated with confounding factors were removed after searching Phenoscanner database. | | | | | | | | | | | | | | | |
