# Supplementary material for: Genetically predicted serum Pimelylcarnitine mediates the association between CD39+ secreting Treg cells and intervertebral disc degeneration
Source: Medicine (Baltimore). 2026 May 15;105(20):e48540. doi: 10.1097/MD.0000000000048540 (PMC13183168; doi:10.1097/MD.0000000000048540)
Supplement: Supplementary file 5 [file medi-105-e48540-s005.docx]

Supplementary Figure 1 Scatter plots in the present Mendelian randomization study

(A) Scatter plot of the causal effects of CD39^+^ secreting Treg associated SNPs on IVDD

(B) Scatter plot of the causal effects of CD39^+^ secreting Treg associated SNPs on C7-DC

(C) Scatter plot of the causal effects of C7-DC associated SNPs on IVDD

| 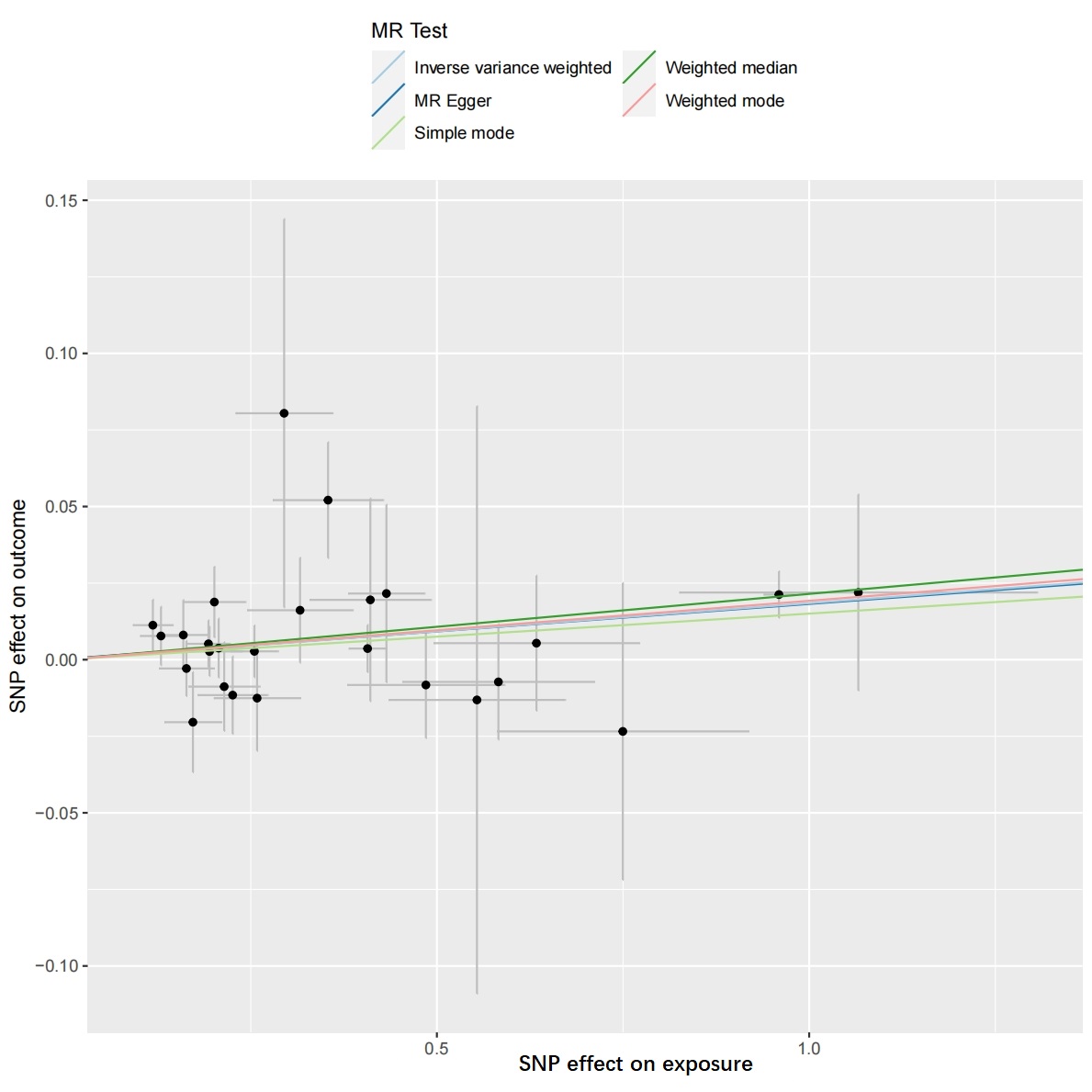  （A） | 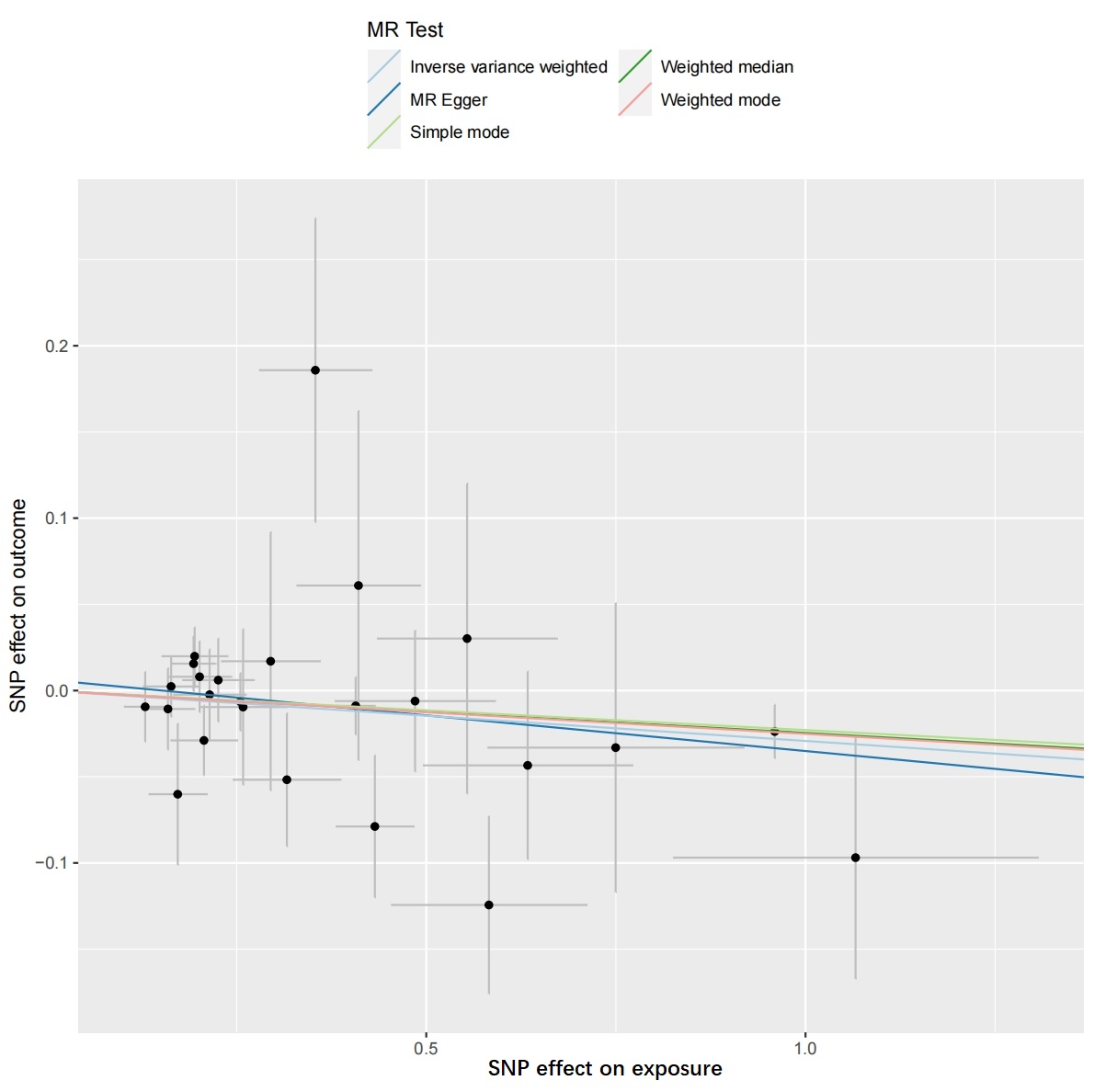  （B） | 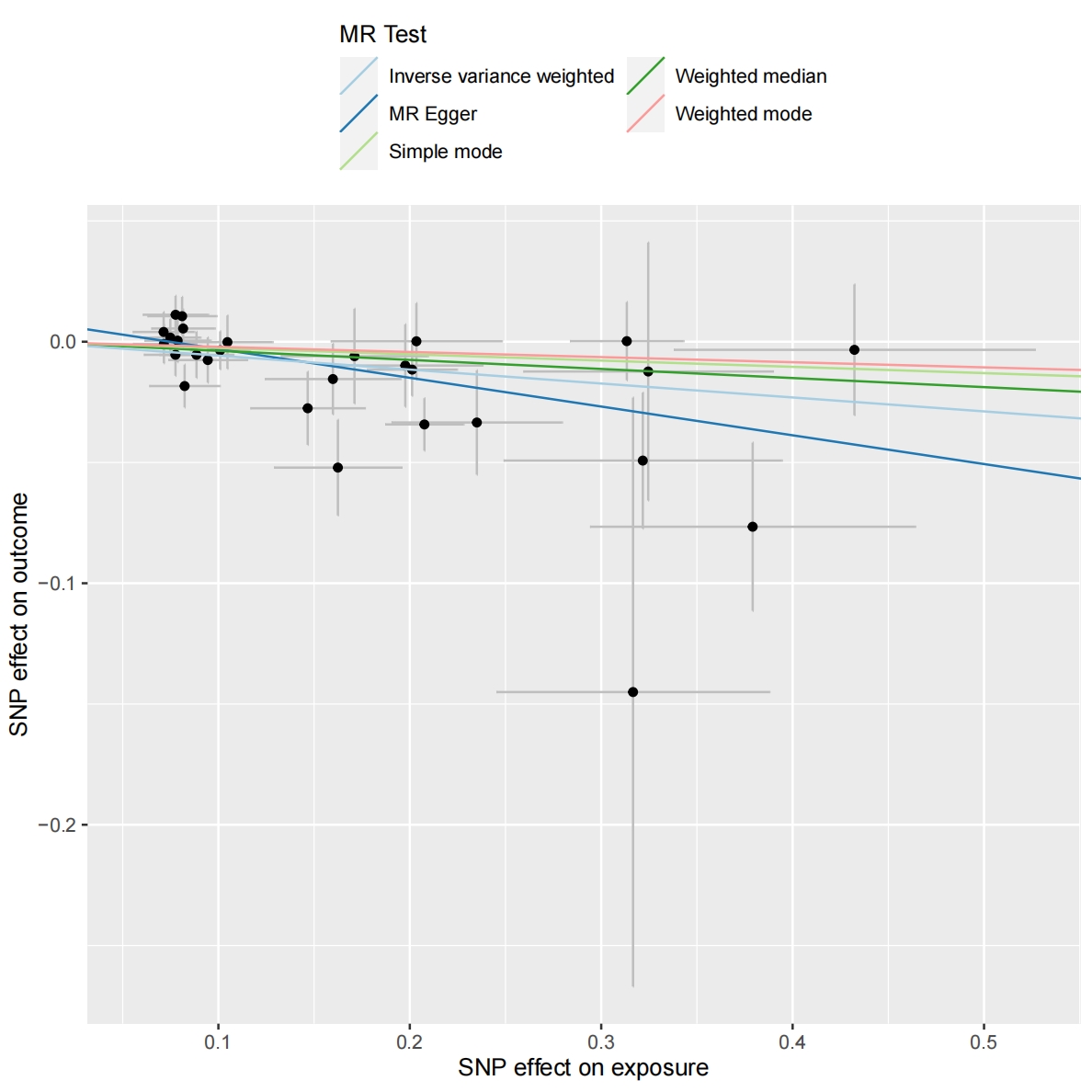  （C） |
| --- | --- | --- |
